# Clustering of Spontaneous Recurrent Seizures in a Mouse Model of Extended Hippocampal Kindling

**DOI:** 10.3389/fneur.2021.738986

**Published:** 2021-11-25

**Authors:** Haiyu Liu, Liang Zhang

**Affiliations:** ^1^Department of Neurosurgery, The First Hospital of Jilin University, Changchun, China; ^2^Krembil Research Institute, University Health Network, Toronto, ON, Canada; ^3^Division of Neurology, Department of Medicine, University of Toronto, Toronto, ON, Canada

**Keywords:** mice, EEG, spontaneous recurrent seizures, kindling, hippocampus, epilepsy

## Abstract

Acute repetitive seizures or seizure clusters are common in epileptic patients. Seizure clusters are associated with a high risk of developing status epilepticus and increased morbidity and mortality. Seizure clusters are also recognizable in spontaneous recurrent seizures (SRS) that occur in animal models of epilepsy. The electrical kindling of a limbic structure is a commonly used model of temporal lobe epilepsy. Although classic kindling over the course of a few weeks does not generally induce SRS, extended kindling over the course of a few months can induce SRS in several animal species. SRS in kindled cats often occur in clusters, but the existence of seizure clusters in rodent models of extended kindling remains to be demonstrated. We explored the existence of seizure clusters in mice following extended hippocampal kindling. Adult male mice (C57BL/6) experienced twice daily hippocampal stimulations and underwent continuous 24-hour electroencephalogram (EEG)-video monitoring after ≥80 stimulations. SRS events were recognized by EEG discharges and associated motor seizures. Seizure clusters, defined as ≥4 seizures per cluster and intra-cluster inter-seizure intervals ≤ 120 min, were observed in 19 of the 20 kindled mice. Individual mice showed variable seizure clusters in terms of cluster incidence and circadian-like expression patterns. For clusters consisting of 4–7 seizures and intra-seizure intervals ≤ 20 min, no consistent changes in inter-seizure intervals, EEG discharge duration, or motor seizure severity scores were observed approaching cluster termination. These results suggested that seizure clustering represents a prominent feature of SRS in hippocampal kindled mice. We speculate that, despite experimental limitations and confounding factors, systemic homeostatic mechanisms that have yet to be explored may play an important role in governing the occurrence and termination of seizure clusters.

## Introduction

Many patients with epilepsy experience acute repetitive seizures or seizure clusters, which are associated with shorter-than-typical inter-seizure intervals (1–5). Various definitions of seizure clusters have been suggested, such as 2–4 seizures within 24–48 h, 2–3 generalized or complex partial seizures within 4 h, a three-fold to four-fold increase over the patient's typical seizure frequency, or multiple seizures with distinct patterns from the patient's usual seizure pattern that occur within 12–24 h [see review by (2)]. Seizure clusters impose considerable negative impacts on the quality of life for both patients and caregivers. Epileptic patients with seizure clusters are at higher risk of developing status epilepticus (SE) and experience increased morbidity and mortality compared with non-clustering epilepsy patients. The non-intravenous administration of benzodiazepines as rescue medications is recommended for seizure clusters. The pathogenic mechanisms underlying seizure clusters are not well understood, but some risk factors have been recognized such as extra temporal or frontal lobe epilepsy, SE, refractory epilepsy, poor seizure control and increased duration of epilepsy. Certain behavioral changes such as changes in sleep disruption, stress, and medication changes or non-compliance can also heighten individual vulnerability to seizure clusters (2–5). Chronic electroencephalogram (EEG) monitoring in individual epileptic patients has demonstrated the existence of diverse timescales for seizure occurrence, with rhythmic patterns that can be daily (circadian), multi-day (multidien), or yearly (circannual) (6–8). Seizure clusters may result from discrete patterns that have yet to be recognized in individual patients, and further unraveling of seizure occurrence patterns may improve the management of epileptic seizures.

Previous studies have observed seizure clusters in rodent models of epilepsy. In these models, spontaneous recurrent seizures (SRS) were induced by the intraperitoneal injection of pilocarpine (9–12) or kainite (12–14), by the intra-cortical (15) or intra-hippocampal (16) application of tetanus toxin, or by the neonatal induction of hypoxia-ischemia (17). SRS often appeared in clusters, encompassing multiple seizures over the course of 1–3 days, and displayed seizure-free or seizure-infrequent inter-cluster periods that lasted for a few days in most cases. SRS occurrence with circadian patterns was also noticed in individual animals. It is conceivable that animal model studies may help understand biological mechanisms of seizure clusters therefore providing relevant information for seizure managements in clinical settings.

Kindling, which refers to the repetitive electrical stimulation of a limbic structure, is a widely used model of temporal lobe epilepsy (18–21). Although the classic kindling protocol, which typically lasts for a few weeks, does not generally induce SRS [but see (22)], extended kindling over the course of a few months can induce SRS. Specifically, the extended kindling of the amygdala, perforant pathway, prefrontal cortex, hippocampus, or olfactory bulb successfully induced SRS in monkeys (23, 24), dogs (25), cats (26–29), rats (30–34), and mice (35). The clustering of SRS was observed in amygdala-kindled cats, with more than two seizures occurring in each cluster, and the shortest inter-seizure interval was reported as 30 min (29). Thus far, no reports have described seizure clusters in rodent models of extended kindling. We attempted to provide information in this area using a mouse model of extended hippocampal kindling that was developed in our laboratory (36–38). Considering that temporal lobe epilepsy is highly diverse in etiologies and electro-clinical manifestations (39, 40), our present observations may complement the previous studies (see references above) that characterize seizure clusters in other models of temporal lobe epilepsy.

## Materials and Methods

### Animals

Male C57 black mice (C57BL/6N; Charles River Laboratory, Saint-Constant, Quebec, Canada) were used in this study. All mice were housed in standard cages with food and water *ad libitum* and were maintained in a local vivarium that was maintained at temperatures between 22 and 23°C with a 12-h light-on/off cycle (light-on starting at 6:00 am). Aged C57BL mice (≥24 months) often encounter health-related complications, including skin lesions, ear infections, and tumors. Therefore, we chose to kindle mice 11–13 months old to model new-onset temporal lobe epilepsy, similar to that observed in adult and aging populations (40) while minimizing the potential occurrence of health-related complications that are common in aged mice (41). All experiments conducted in this study were reviewed and approved by a local animal committee (University Health Network Animal Use Protocol #986.40) in accordance with the Guidelines of the Canadian Council on Animal Care.

### Electrode Implantation, Hippocampal Kindling, and EEG Recording

Electrode implantation and extended kindling were performed, as previously described (36–38). Each mouse was implanted with two pairs of twisted-wire bipolar electrodes that were made of polyimide-insulated and tip-exposed stainless-steel wires (outer diameter 0.125 mm; Plastics One, Virginia, USA). One electrode pair was positioned at the hippocampal CA3 region to provide kindling stimulation and local recordings, and the second pair was positioned at an ipsilateral or contralateral site, according to group. Five groups of mice were established, targeting the contralateral hippocampal CA3 [bregma: −2.5 mm, lateral: 3.0 mm, and depth: 3.0 mm; (42)], the contralateral or ipsilateral parietal cortex (bregma: −0.5 mm, lateral: 2.0 mm, and depth: 0.5 mm), the ipsilateral piriform cortex (bregma: 0.5 mm, lateral: 3.0 mm, and depth: 5.0 mm), the contralateral dorsal-medial thalamus (bregma: −1.5 mm, lateral: 0.5 mm, and depth: 3.5 mm), or the ipsilateral entorhinal cortex (bregma: −3.5 mm, lateral: 4.0 mm, and depth: 5.0 mm). A reference electrode was positioned at a frontal area (bregma: +1.5 mm, lateral: 1.0 mm, and depth: 0.5 mm). We used this implantation approach to explore regional EEG discharges in the kindled CA3 and a non-stimulated brain area in individual mice while minimizing the potential complications associated with multi-electrode implantations in the small mouse brain. The unstimulated structures were selected according to previous studies of extended kindling performed in monkeys and cats (23, 24, 26, 29). The putative tip locations of the implanted electrodes were determined later by histology, as described in our previous study (38).

We used a train of stimuli (60 Hz for 2 seconds) for hippocampal kindling (35, 36, 38, 43–45). Briefly, constant current pulses (monophasic waveforms with pulse duration of 0.5 ms and current intensities of 10–150 μA) were generated by a Grass stimulator and delivered through a photoelectric isolation unit (model S88, Grass Medical Instruments, Warwick, Rhode Island, USA). Stimulations with incremental intensities were used to determine the threshold of evoked after-discharges in individual mice. Kindling stimulations were conducted at 25% above the threshold value. Hippocampal kindling stimulations were applied twice daily, ≥5 h apart (30–34, 36). Each stimulation episode lasted for a few min while the mouse was placed in a glass container for EEG-video monitoring (35, 45).

EEG-video monitoring for 24 h was performed after 80, 100, 120, or 140 stimulations. Kindling stimulations were terminated if mice exhibited ≥2 SRS events within a 24-h monitoring period. SRS events were observed in individual mice following 80–140 stimuli. SRS development and SRS expression were largely comparable in mice with different electrode implantation schemes (38). Therefore, all data from mice with electrodes implanted at different positions were pooled in the present analysis.

Local differential recordings using the twisted-wire bipolar electrodes were used to monitor EEG activity (38). Signals were collected using two-channel or one-channel microelectrode amplifiers with extended head-stages (model 1800 or 3000, AM Systems; Sequim, Washington, USA). These amplifiers were set with an input frequency band of 0.1–1,000 Hz and an amplification gain of 1,000. Amplifier output signals were digitized at 5,000 Hz (Digidata 1440A or 1550, Molecular Devices; Sunnyvale, California, USA). Data acquisition, storage, and analyses were conducted using pClamp software (Version 10; Molecular Devices).

### Continuous EEG-Video Monitoring

Continuous 24-h EEG-video monitoring was performed, as previously described (35–38). Briefly, each mouse was placed in a modified cage with food and water *ad libitum*. A slip ring commutator was mounted atop the cage and connected to the implanted electrodes via flexible cables for EEG recording. A webcam (model C615, Logitech, Canada) was placed near the cage to capture animal motor behaviors. Video was acquired at 20–25 frames per second. Dim lighting was used to facilitate webcam monitoring during the light-off period. A cursor auto-click program (Mini Mouse Macro program; http://www.turnssoft.com/mini-mouse-macro.html) was used to manage concurrent EEG and video recordings and to save data every 2 h. EEG and video data were collected approximately 24 h each day for up to 7 consecutive days per recording session. As necessary, EEG-video monitoring was stopped for 10–30 min in the morning to allow for cage cleaning, the addition of food and water, or other animal care procedures.

### SRS Detection

SRS were detected by EEG discharges and associated motor seizures. Spontaneous EEG discharges were recognized as repetitive spikes waveforms with amplitudes ≥2-fold the background signals and durations ≥10 seconds. Nearly all discharges displayed low-voltage fast signals at the onset. Discharge termination, in most cases, featured a sudden cessation of spike activity and a subsequent signal suppression component lasting several seconds. Discharge durations were estimated as the time between the onset of low-voltage fast signals and the cessation of spike activity (38). Discharge events were inspected independently by three researchers. Uncertain events associated with spontaneous discharges (≤ 2% of SRS events in individual mice) were not included in the present analysis [see (38)].

The Racine scale modified for mice (18, 43) was used to evaluate the severity of spontaneous motor seizures. Briefly, stage 0 indicated no response or behavioral arrest; stage 1 represented chewing or facial movement; stage 2 was characterized by chewing, head nodding, or unilateral forelimb clonus; stage 3 featured bilateral forelimb clonus; stage 4 was associated with rearing behavior; and stage 5 indicated a fall or the loss of righting reflex. Spontaneous motor seizures were assessed independently by seven researchers through video reading (38). The concordance rates for recognizing stage 3–5 seizures were ≥90% among these researchers.

SRS occurrence was detected by combined EEG and video inspections. EEG signals were first screened to detect spontaneous discharges. Detected discharge events were time-stamped, and the corresponding video data were reviewed to score motor seizures. Discharge and motor seizure assessments were performed separately, as mentioned above. We used this approach for convenient SRS detection because spontaneous discharges with large amplitudes and co-expression in both kindled and unstimulated sites were clearly distinguishable from background signals in our EEG data, whereas the scoring of motor seizures through video reading is laborious and often complicated by the mouse's position within the cage or surrounding bedding materials. Due to these complications encountered during video reading, we did not analyze motor seizures by video reading alone. In the present analysis, SRS were characterized according to the recognition of EEG discharges, with or without associated motor seizures, due to errors encountered in video acquisition and storage during the study.

### Data Selection and Statistical Analysis

Data from 20 kindled mice were included in the present analysis. In these mice, continuous 24-h EEG-video monitoring was reliable for 4–7 consecutive days per session and for up to 3 sessions. All SRS events detected from these monitoring sessions were analyzed. Data collected via continuous 24-h EEG-video monitoring of 1–3 days were not included in the present analysis expect where specified. Such data selection was designed to minimize the confounding factors associated with potential disturbances or stress associated with EEG monitoring or changes in the cage setting (see Discussion).

Statistical tests were conducted using Origin software (Northampton, MA, USA). Data are presented as the mean and the standard error of the mean (SEM) throughout the text and figures. Statistical significance was set to *p* < 0.05. For normally distributed data, group differences were assessed using a Student's *t*-test or one-way analysis of variance (ANOVA), followed by a Bonferroni *post-hoc* test. When data were not distributed normally, a Mann–Whitney U test or a non-parametric rank-based ANOVA (Kruskal–Wallis), followed by a *post hoc* test, was used for group comparisons.

## Results

### Hippocampal Kindling and SRS Detection

**Figure 1:**
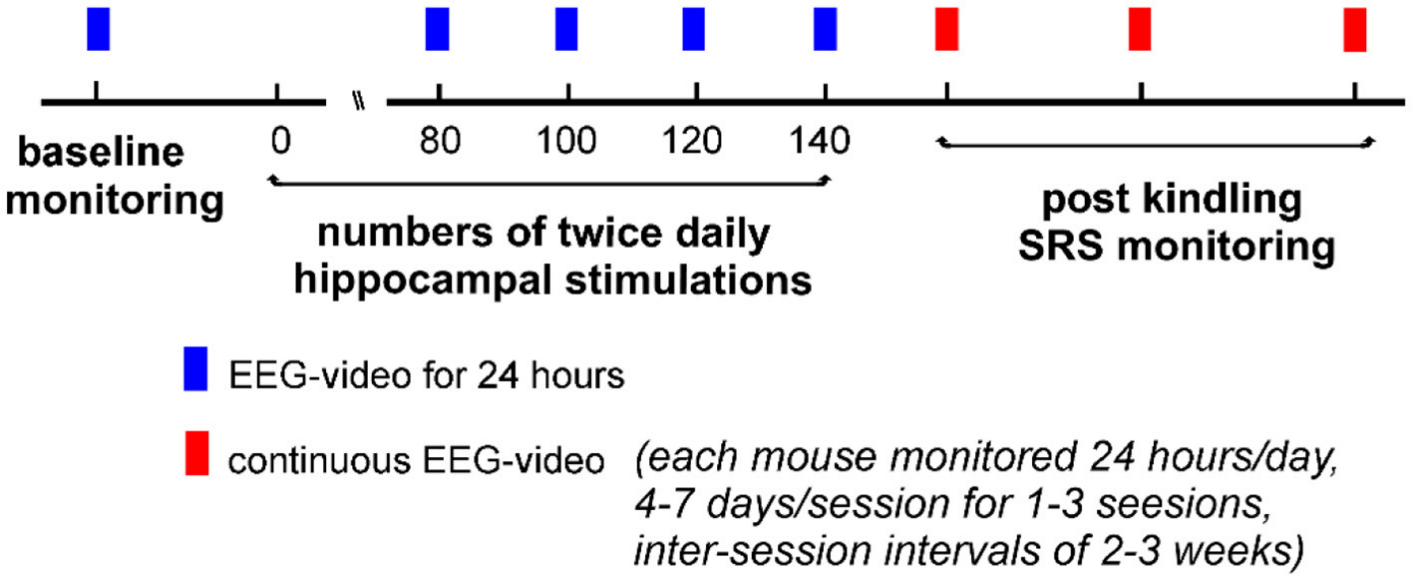
A schematic outline of the experimental protocols. Individual mice experienced twice daily hippocampal stimulations for up to 140 stimulations. Electroencephalogram (EEG)-video monitoring for 24 h was performed before and after 80, 100, 120, or 140 stimulations. No stimulation was applied if ≥2 SRS events were detected within a single 24-h monitoring period. Continuous 24-h EEG-video monitoring (4–7 days per session) was performed after the termination of kindling stimulation. Similar EEG-video monitoring was repeated 2–3 times if suitable (2–3 weeks apart between sessions).

Our experimental protocols are schematically outlined in Figure 1. Briefly, individual mice received hippocampal kindling stimulations twice daily and underwent EEG-video monitoring for 24 h after 80, 100, 120, or 140 stimulations. No further kindling stimulation was applied if ≥2 SRS were detected within a single 24-h monitoring period. Continuous 24-h EEG-video monitoring was performed for up to 7 consecutive days after the termination of kindling stimulation (the first monitoring session). Continuous EEG-video monitoring was resumed 2–3 and 4–6 weeks later (the second and the third monitoring sessions), if suitable.

**Figure 2:**
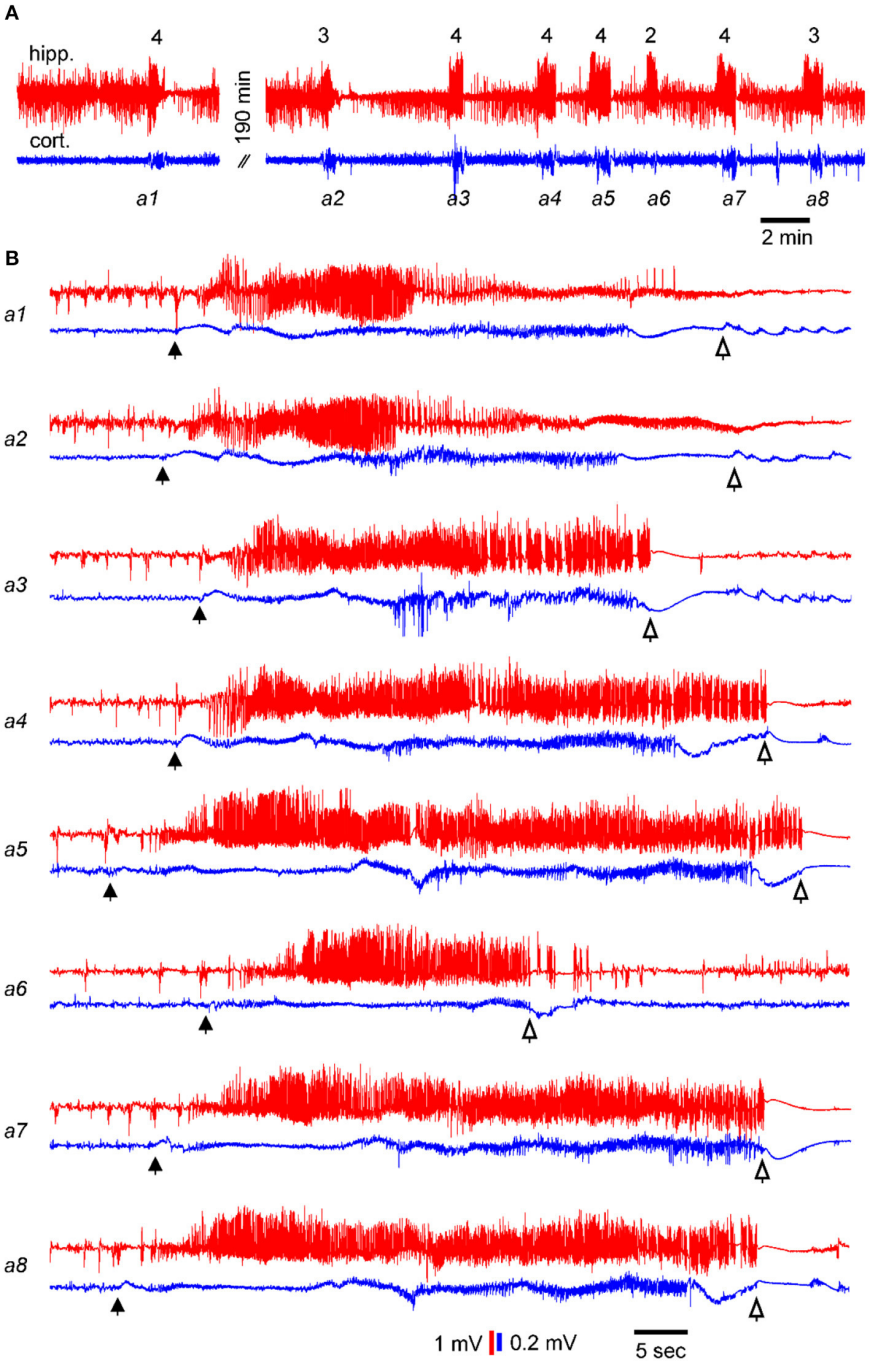
Examples of consecutive SRS events. Data showing electroencephalogram (EEG) signals recorded simultaneously from the kindled hippocampus (red) and ipsilateral parietal cortex (blue) of a single mouse. EEG signals were collected in a wide-frequency band (0.1–1000 Hz). **(A)** EEG traces showing 8 consecutive discharges. Corresponding motor seizure scores are indicated above the hippocampal discharges. **(B)** Sequential discharges were expanded. Filled and open arrows denote putative discharges onset and termination points, respectively. Note, in A, the first and second discharges were approximately 190 min apart, whereas the inter-seizure intervals of subsequent seizures were 2–6 min.

SRS were recognized by EEG discharges and associated motor seizures (35, 38). An example showing eight consecutive discharges is illustrated in Figure 2. Discharges were recorded simultaneously from the kindled hippocampus and the ipsilateral parietal cortex (Figure 2A). Motor seizure scores were assessed according to the 5-stage Racine scale modified for mice (18, 43), and the corresponding scores are indicated above the EEG traces (Figure 2A). Individual events from the eight consecutive discharges were expanded (Figure 2B), and the putative discharge onset and termination points were indicated for concurrent regional discharges. Inter-seizure intervals (ISIs), as measured according to the discharge timings in the EEG recordings, were approximately 195 min between the first and second seizures and 2–6 min between the subsequent 7 seizure events. ISIs were similarly estimated for other SRS events included in the present analysis.

### Inter-seizure Intervals (ISIs)

SRS data collected from 20 kindled mice were analyzed. EEG electrodes were implanted in the hippocampal areas and the ipsilateral/contralateral cortical areas (nine mice); the hippocampal and contralateral thalamic areas (five mice); bilateral hippocampal areas (three mice); hippocampal and ipsilateral piriform regions (two mice); or hippocampal and ipsilateral entorhinal cortical areas (one mouse). Because the EEG discharges and corresponding motor seizures were largely comparable across mice with electrodes implanted in different regions (38), SRS collected from all 20 mice were pooled for analysis. Overall, a total of 1,243 ISI estimates were obtained from the 20 mice through continuous 24-h EEG-video monitoring (4–7 consecutive days per session and up to 3 sessions). ISIs ranged from 2–1,226 min, and 22–77 ISI estimates were obtained in the first monitoring session for each individual mouse.

**Figure 3:**
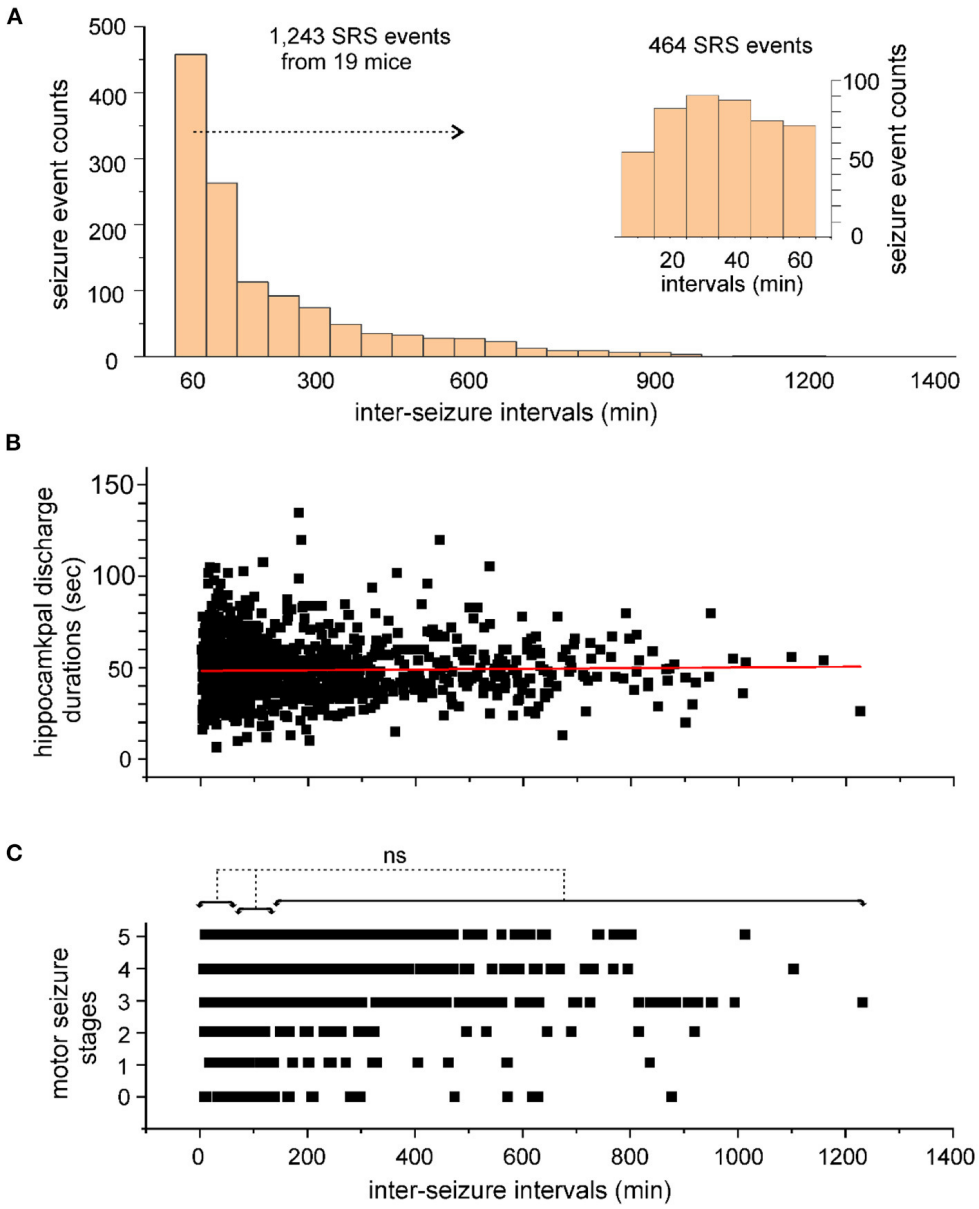
Inter-seizure intervals of SRS events. **(A)** Inter-seizure intervals (ISIs) of SRS events were estimated in 20 kindled mice. A total of 1,243 ISI estimates were binned every 60 min and presented in a histogram. ISIs ≤ 60 min were further separated and binned every 10 min (A, inset). **(B,C)** Discharge durations of the kindled hippocampus **(B)** and corresponding motor seizure scores **(C)** were plotted against the ISIs. The red line through the data points in B shows the outcome of Pearson's correlation analysis (*r* = 0.025). No significant difference (ns, *p* > 0.05, nonparametric ANOVA) among motor seizure scores in C was identified among ISIs with lengths ≤ 60 min, between 60 and 120 min, or between 120 and 1,226 min.

An ISI histogram was generated to view the overall distribution of these estimates (Figure 3A). When ISI estimates were binned every 60 min, 464 ISIs ≤ 60 min and 261 ISIs lasting 60–120 min were identified, accounting for 37 and 21% of total ISI estimates, respectively. The 464 ISIs ≤ 60 min were further examined (Figure 3A insert) using 10-min bins, resulting in the identification of 54–89 ISIs in each bin.

To explore a potential overall relation between seizure severity and ISI length, the durations of the kindled hippocampal discharges and corresponding motor seizure scores were plotted against seizures with different intervals from preceding events (Figures 3B,C). Hippocampal discharges of variable durations were observed for seizures detected at different times after preceding events, with no strong correlation between these two measures (Pearson's correlation analysis, *r* = 0.025; Figure 3B). Stages 1–5 motor seizures were similarly observed for seizures detected at different times after preceding events. Motor seizures detected ≤ 60 min, between 60 and 120 min, or between 121 and 1,226 min after preceding events, were scored as 3.3 ± 0.04 (*n* = 372), 3.2 ± 0.08 (*n* = 212) or 3.4 ± 0.06 (*n* = 375), respectively, and no significant difference among these three measures was detected (*p* > 0.05; nonparametric ANOVA; Figure 3C). Together, these observations suggest that a large portion of SRS events occur with ISIs ≤ 120 min and that the SRS severity is generally independent of the ISI in hippocampal kindled mice.

### Seizure Clusters

Previous studies have characterized seizure clusters in other rodent models of epilepsy (9–16). In these models, seizure clusters were generally comprised of multiple seizure events that were observed over 1–3 days and separated by seizure-free or seizure-infrequent periods lasting for a few days. In our previous experiments, we observed 6-14 SRS events per day from individual kindled mice (35, 38). In our present analysis, 58% of ISI estimates were ≤ 120 min (Figure 3A). Based on the above information, we defined seizure clusters in our model as ≥4 consecutive seizures with intra-cluster ISIs ≤ 120 min. Seizure clusters were recognized in SRS data collected from 19 of the 20 mice analyzed. No seizure clusters were detected in the remaining mouse with 29 SRS events collected over 4 consecutive days.

**Figure 4:**
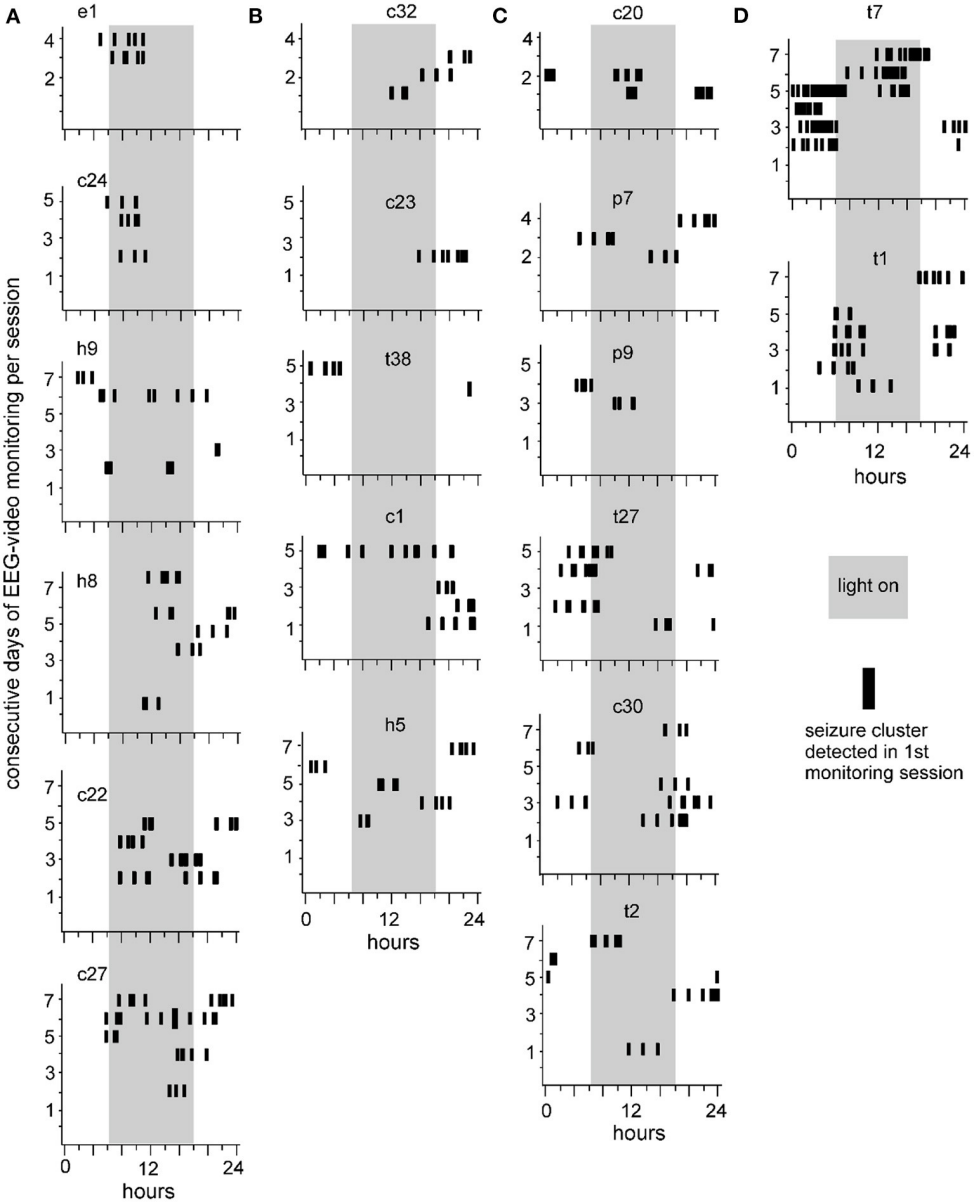
Seizure clusters recognized during the first session of 24-h EEG-video monitoring. Data collected from 19 mice are presented in individual plots. Mice are indicated by small letters and numbers, with “c, e, h, p, or t” standing for unstimulated cortical, entorhinal, hippocampal, piriform, or thalamic area, respectively. Y-axis – consecutive days of 24-h EEG-video performed in individual mice. X-axis – 24 h of the day. Long gray bars – the light-on period (6 am to 6 pm). Short black vertical bars – seizure clusters recognized at times of consecutive days. **(A,B)** Seizure clusters were observed largely in the light-on **(A)** or light-off **(B)** period. **(C)** Seizure cluster expressions in both the light-on and light-off periods. **(D)** Seizure clusters with relatively confined expression in the light-on or light-off period.

Seizure clusters recognized in the first monitoring session are presented in Figure 4. Daily seizure clusters were plotted for 19 individual mice. The consecutive days of 24-hour EEG-video performance are indicated for each mouse (Y-axis). Seizure cluster expressions at different times of day (0–24 h, X-axis) are denoted by black vertical bars. Three trends were noted in seizure cluster expression. First, most seizure clusters occurred in groups, with 2–7 clusters per group, and neighboring groups occurred 3–26 h apart. Second, the incidence of seizure clusters was variable across individual mice. Four mice showed 1–2 groups of seizure clusters while being monitored for 4–5 consecutive days (e1 in A, c23 and t38 in B, and p9 in **C**), whereas the other mice displayed multiple groups of seizure clusters each day for several consecutive days. Third, seizure clusters presented variable circadian-like patterns. Specifically, seizure clusters were more frequently observed in the light-on than in the light-off period for 6 mice (18.4 ± 4.4 vs. 7.2 ± 2.2 events, *p* = 0.046; Figure 4A) and mainly observed in the light-off relative to the light-on period for 5 mice (15.8 ± 3.5 vs. 5.2 ± 1.9 events, *p* = 0.028; Figure 4B). Seizure clusters occurred at similar rates during both the light-on and light-off periods for 8 mice (20.9 ± 7.8 and 18.1 ± 6.7 events, *p* = 0.635; Figures 4C,D), including in relatively confined periods for 2 mice (t7 and t1 in Figure 4D).

**Figure 5:**
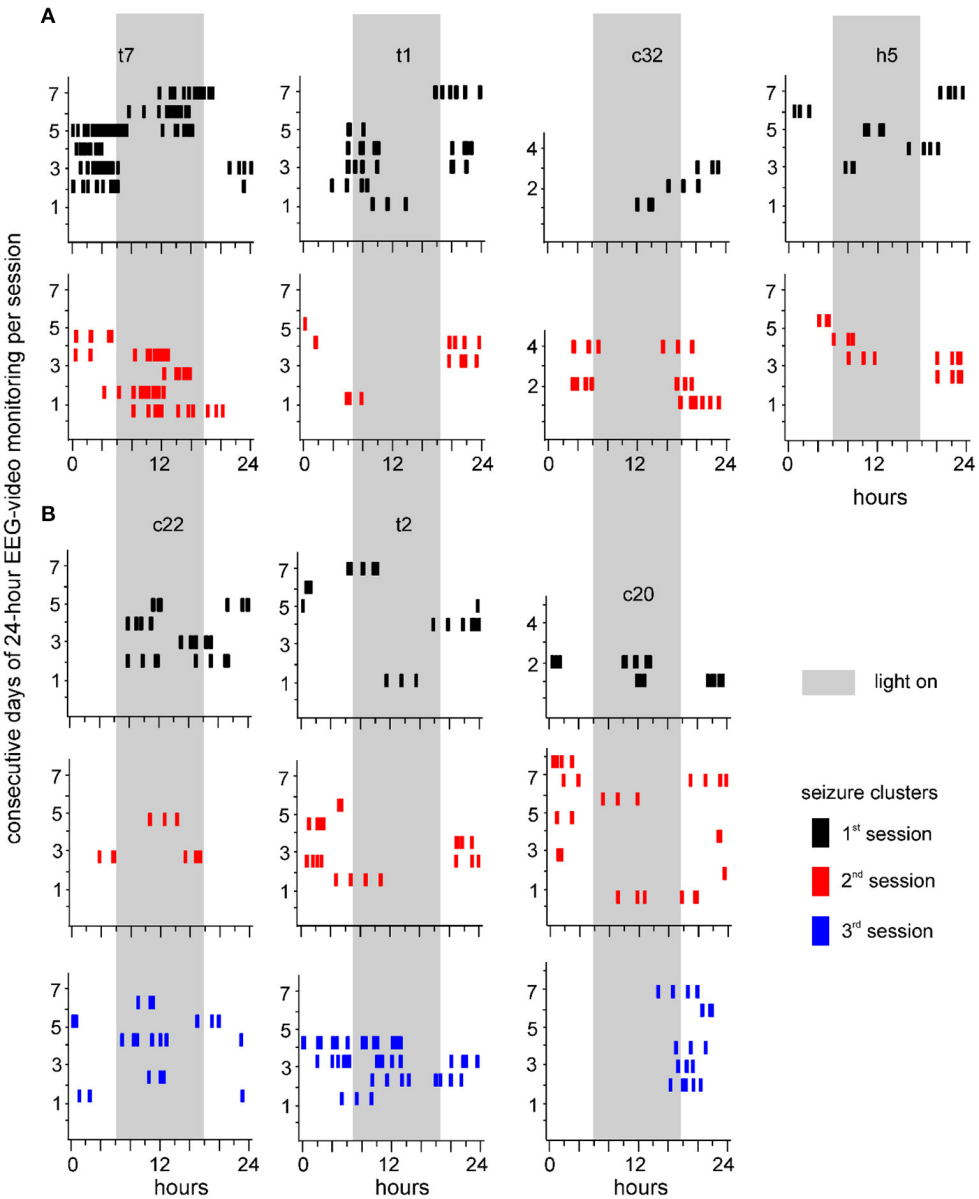
Seizure clusters recognized during 3 sessions of 24-h EEG-video monitoring. Seizure clusters recognized from 7 individual mice over 2 **(A)** or 3 **(B)** monitoring sessions are presented in each plot. Mice are named by small letters and numbers, with “c, h, or t” standing for unstimulated cortical, hippocampal, or thalamic area, respectively. Y-axis – consecutive days of 24-h EEG-video performed in individual mice. X-axis – 24 h of the day. Long gray bars – the light-on period (6 am to 6 pm). Seizure clusters detected in the first, second, or third sessions are denoted by black, red, or blue vertical bars, respectively. Note persistent but variable seizure cluster expressions in individual mice.

Seizure clusters were observed in 7 of 19 mice over 2–3 monitoring sessions (Figure 5). Sequential sessions were separated by 2–3-week intervals. Seizure cluster expressions per session were plotted for individual mice. Although seizure clusters were observed in repeated monitoring sessions, their expressions over time varied considerably in individual mice. For example, seizure cluster expression across all 3 sessions primarily occurred during the light-on period for one mouse (c22 in Figure 5B). By contrast, seizure clusters with relatively confined expression during the third session were noticeable in two mice (t2 and c20 in Figure 5B). Seizure clusters were also observed in 8 of 19 mice that underwent repeated 24-h EEG-video monitoring for 1–3 days and 3–9 weeks after termination of kindling stimulation (data not shown). Together, these observations suggest that seizure clusters represent an enduring feature of SRS in extended kindled mice.

### Intra-cluster Seizure Severities and ISI

**Figure 6:**
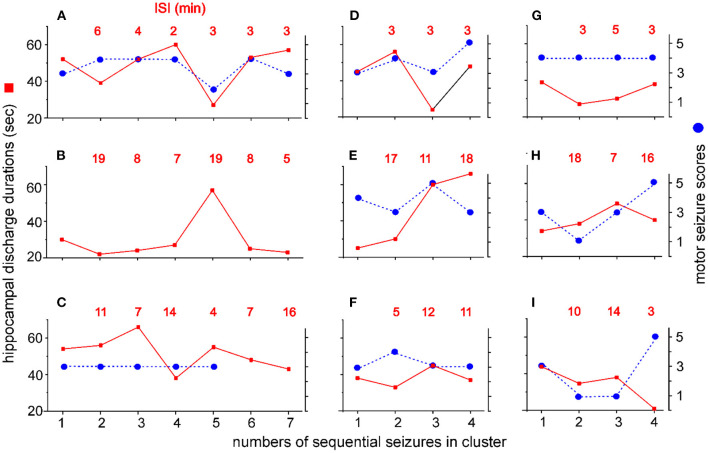
Intra-cluster seizure severities and ISIs. Seizure clusters with ≥4 seizures per cluster and intra-cluster ISIs ≤ 20 min were observed from 9 mice. Y axis - discharge durations of the kindled hippocampus (red squares, left) and corresponding motor seizure scores (blue circles, right). X axis - numbers of sequential seizures in cluster. Intra-cluster ISIs are indicated by red letters in each plot. Data were plotted for individual mice **(A**–**I)**. Motor seizure scores corresponding to the discharges in **(B)** and to the last two discharges in **(C)** could not be determined due to errors in the video recordings. Note no consistent increase or decrease in discharge durations, motor seizure scores, or ISIs approaching cluster termination.

Seizure clusters that encompassed ≥4 seizures with ISIs < 20 min (*n* = 9 clusters, one per mouse) were detailed to explore potential patterns in intra-cluster seizures. The discharge durations of the stimulated hippocampus, scores of corresponding motor seizures, and intra-cluster ISIs were plotted against the sequential seizures in each cluster (Figure 6). The discharge durations, corresponding motor seizure scores, and intra-cluster ISIs were variable in individual clusters, and no consistent decrease or increase in these three parameters was observed approaching cluster termination for any of the 9 clusters examined. These observations suggest that systemic homeostatic mechanisms or factors rather than local forebrain circuitry activity may underlie the genesis and termination of seizure clusters in kindled mice (see Discussion).

## Discussion

The primary goal of our present study was to explore the existence of seizure clusters in SRS observed in hippocampal kindled mice. Our analysis resulted in four primary observations. (1) More than half of SRS events occurred with ISIs ≤ 120 min. SRS severity, as determined by hippocampal discharge durations and associated motor seizure scores, was independent of ISI length. We defined seizure clusters in our mouse model as ≥4 consecutive seizures with intra-cluster ISIs ≤ 120 min. (2) Seizure clusters were defined in SRS events collected from 19 of 20 mice examined. Seizure clusters were persistent, observed in mice that underwent repeated EEG-video monitoring for up to 9 weeks after the termination of kindling stimulation. (3) Seizure clusters were variable in individual mice in terms of both incidence and circadian-like patterns. (4) Seizure severities and ISI lengths within clusters showed no consistent changes approaching cluster termination. Our present observations are in line with a previous study in extended kindled cats (29) and suggest that seizure clusters may be a noteworthy feature of SRS in rodent models of extended kindling.

Seizure clusters have been observed in other rodent models of epilepsy, including SRS induced by the intraperitoneal injection of pilocarpine (9–12, 46) or kainite (12–14), the intra-cortical (15) or intra-hippocampal (16) injection of tetanus toxin, or neonatal hypoxia-ischemia (17). Seizure clusters in these models are comprised of multiple seizures that occur over 1–3 days, and neighboring clusters are separated by seizure-free or seizure-infrequent periods that last for a few days. Circadian patterns in SRS events or seizure cluster occurrence are recognizable in individual animals. The seizure clusters we defined in kindled mice differed from those in previous studies in terms of both the number of seizures per cluster and the inter-cluster intervals. This difference may be partly attributable to the more frequent SRS events observed in hippocampal kindled mice compared with other SRS induction methods. In our experiments, 6–14 SRS events per day were generally observed in individual mice, and SRS-free 24-h periods were rarely observed (35, 38). However, there were limitations in our continuous 24-h EEG-video monitoring, which might fail to reveal SRS-free 24-h periods that might occur sporadically or infrequently in individual mice.

In rats with SRS induced by intra-hippocampal injections of tetanus toxin, seizure clusters were defined as a group of at least 10 seizures separated by an ISI no longer than 12 h and lasting no more than 96 h (16). Within each cluster, a progressive increase in ISI was observed approaching cluster termination. The lengthening of the intra-cluster ISI was paralleled by increasing behavioral seizure severity, the occurrence of convulsive seizures, the recruitment of extra-hippocampal structures, and the spread of electrographic epileptiform activity outside of the limbic system. Focal (non-convulsive) seizures that occurred at the beginning of the cluster were hypothesized to act via positive feedback, contributing to cluster progression and aggravating successive seizure activity. The subsequent occurrence of convulsive seizures provides negative feedback to progressively decrease the seizure rate toward cluster termination, followed by a long seizure-free period (16). No overall relation between seizure severity and ISI was observed in our model; therefore, seizure clusters that encompassed 4–7 seizures and had intra-cluster ISIs ≤ 20 min were analyzed to reveal intra-cluster changes. No consistent increases in the intra-cluster ISI, hippocampal discharge duration, or corresponding motor seizure scores were observed in any of the seizure clusters analyzed. These observations suggest that changes in intra-cluster seizures are not a major determining or influencing factor in the cluster termination of kindled mice. The mechanisms underlying SRS genesis and seizure clusters may differ between the rat model of intra-hippocampal injection of tetanus toxin and the mouse model of hippocampal kindling.

Our previous work showed that SRS featured concurrent EEG discharges in multiple forebrain structures and the EEG discharge duration was unrelated to the severity of associated motor seizures (38). One hypothesis derived from these results is that the discharges involving a macroscopic forebrain network can initiate SRS events, but the forebrain discharges may not directly control the severity of associated motor seizures (38). Based on both previous work and our present observations, we speculate that systemic homeostatic mechanisms beyond the proposed forebrain network activity may play a key role in governing the occurrence and termination of seizure clusters in hippocampal kindled mice.

Overall, our present analysis suggest that seizure clusters are significant and enduring features of SRS events in the mouse model of extended hippocampal kindling, but seizure clusters are variable in individual mice in terms of cluster frequencies and circadian-like expression patterns. Although such variability appears to be in line with previous studies in other rodent models of epilepsy (9–16), multiple limitations and complications associated with our experimental conditions remain confounding factors. In our experiments, each mouse was placed in a modified cage for continuous EEG-video monitoring and tethered EEG signals were collected with the aid of a commutator mounted atop the cage (36). Although the mouse was able to move freely in the modified cage during recording, this style of monitoring was stressful for the mouse under recording conditions. In addition, continuous EEG-video monitoring was not isolated from environmental noises, and dim lighting was necessary to perform webcam monitoring during the light-off period. Such sub-optimal settings might interfere mouse's sleep patterns therefore influencing the timing and/or incidence of seizure clusters. Moreover, continuous EEG-video monitoring (i.e., 24 h per day, 4–7 consecutive days per session and 1–3 sessions per mouse) were successful performed in a limited number of mice, which prevented reliable analysis of circadian patterns of SRS and their changes at various times after termination of hippocampal kindling. Future work with more optimal experimental settings and with continuous monitoring of EEG and electromyograph signals is needed to characterize sleep-arousal cycle related seizure clusters and rhythmic patterns in our model. Despite the limitations and confounds encountered during our experiments, our present analysis of seizure clusters may aid future investigations examining the effects of antiepileptic manipulations in this mouse model.

## Data Availability Statement

The original contributions presented in the study are included in the article/supplementary material, further inquiries can be directed to the corresponding author/s.

## Ethics Statement

The animal study was reviewed and approved by University Health Network Animal Care Committee (Protocol #986.40).

## Author Contributions

HL conducted experiments. HL and LZ performed data analysis. LZ wrote the manuscript. All authors contributed to the article and approved the submitted version.

## Funding

This work was supported by the Epilepsy Research Program of Ontario Brain Institute and the Biology System Program of Natural Science and Engineer Research Council of Canada.

## Conflict of Interest

The authors declare that the research was conducted in the absence of any commercial or financial relationships that could be construed as a potential conflict of interest.

## Publisher's Note

All claims expressed in this article are solely those of the authors and do not necessarily represent those of their affiliated organizations, or those of the publisher, the editors and the reviewers. Any product that may be evaluated in this article, or claim that may be made by its manufacturer, is not guaranteed or endorsed by the publisher.
